# Impact of Aspirin and Clopidogrel Interruption on Platelet Function in Patients Undergoing Major Vascular Surgery

**DOI:** 10.1371/journal.pone.0104491

**Published:** 2014-08-20

**Authors:** Yannick Le Manach, David Kahn, Christilla Bachelot-Loza, Frederic Le Sache, David M. Smadja, Veronique Remones, Marie-Anne Loriot, Pierre Coriat, Pascale Gaussem

**Affiliations:** 1 Departments of Anesthesia & Clinical Epidemiology and Biostatistics, Michael DeGroote School of Medicine, Faculty of Health Sciences, McMaster University, Hamilton, Ontario, Canada; 2 Population Health Research Institute, David Braley Cardiac, Vascular and Stroke Research Institute, Perioperative Medicine and Surgical Research Unit, Hamilton, Ontario, Canada; 3 AP-HP, Hôpital Pitié-Salpêtrière, Department of Anesthesiology and Critical Care, Paris, France; 4 Inserm UMR-S1140, Faculté de Pharmacie, Paris, France; 5 Université Paris Descartes, Sorbonne Paris Cité, Paris, France; 6 AP-HP, Hôpital Européen Georges Pompidou, Service d’Hématologie Biologique, Paris, France; 7 INSERM UMR-S1147, Paris, France; 8 AP-HP, Hôpital Européen Georges Pompidou, Pharmacogénétique et Oncologie Moléculaire, Paris, France; University of Pittsburgh School of Medicine, United States of America

## Abstract

**Aims:**

To investigate functional platelet recovery after preoperative withdrawal of aspirin and clopidogrel and platelet function 5 days after treatment resumption.

**Methods/Results:**

We conducted an observational study, which prospectively included consecutive patients taking aspirin, taking clopidogrel, and untreated controls (15 patients in each group). The antiplatelet drugs were withdrawn five days before surgery (baseline) and were reintroduced two days after surgery. Platelet function was evaluated by optical aggregation in the presence of collagen, arachidonic acid (aspirin) and ADP (clopidogrel) and by VASP assay (clopidogrel). Platelet-leukocyte complex (PLC) level was quantified at each time-point. At baseline, platelet function was efficiently inhibited by aspirin and had recovered fully in most patients 5 days after drug withdrawal. PLC levels five days after aspirin reintroduction were similar to baseline (+4±10%; p = 0.16), in line with an effective platelet inhibition. Chronic clopidogrel treatment was associated with variable platelet inhibition and its withdrawal led to variable functional recovery. PLC levels were significantly increased five days after clopidogrel reintroduction (+10±15%; p = 0.02), compared to baseline.

**Conclusions:**

Aspirin withdrawal 5 days before high-bleeding-risk procedures was associated with functional platelet recovery, and its reintroduction two days after surgery restored antiplaletet efficacy five days later. This was not the case of clopidogrel, and further work is therefore needed to define its optimal perioperative management.

## Introduction

Oral antiplatelet drugs, and particularly aspirin and clopidogrel, are a cornerstone of cardiovascular prevention [1], [2]. Prior to scheduled surgery, anesthesiologists and surgeons are increasingly faced with the choice between drug withdrawal, potential leading to a rebound phenomenon and a risk of thrombosis [3], and drug continuation with its accompanying risk of bleeding. Some operations may be safely conducted without aspirin withdrawal. For example, some cardiac surgery (e.g. coronary artery bypass grafting with cardiac pulmonary bypass) seems to be feasible during ongoing single- or dual-agent antiplatelet therapy [4], [5], [6],[7]. In contrast, antiplatelet drug discontinuation is required before major vascular surgery and neurosurgery.

Current French guidelines on preoperative antiplatelet drug management [8], which are based solely on expert opinion, recommend that clopidogrel be withdrawn five days and aspirin three to five days before high-bleeding-risk procedures. Furthermore, recent data have shown that administration of aspirin before surgery in noncardiac surgical patients and throughout the early postsurgical period had no significant effect on the rate of a composite of death or nonfatal myocardial infarction but increased the risk of major bleeding [9]. These results suggested that thrombosis prevention benefits may be balanced by an increased risk of bleeding. Preoperative optimal management of antiplatelet drugs appeared thus as crucial in procedures as major vascular surgery where high risk of thrombosis and high risk of bleeding co-exist.

Postoperative management of antiplatelet therapy resumption after major surgery is also a concern, given the increased risk of myocardial infarction and ischemic stroke reported after aspirin withdrawal [10]. Pharmacodynamic studies available suggest that a bolus of antiplatelet agents is required to obtain rapidly a protective effect, but this strategy has been conducted out of the perioperative period and the lack of safety studies highly limits the use of this strategy in the postoperative period [10].

The aims of the present study were to evaluate functional platelet recovery after withdrawal of antiplatelet therapy prior to major vascular surgery and the effects of postoperative drug reintroduction.

## Methods

PLANTAGENET study (PLatelets, ANTithrombotic agents, Aortic surgery and GENETics) was an observational study conducted in line with the recently published STROBE guidelines (Strengthening the Reporting of Observational Studies in Epidemiology) [11] with slight adjustments (see below).

### Study Design

Fifteen consecutive patients were prospectively enrolled in each arm (patients treated with clopidogrel or aspirin for at least four weeks, and untreated controls) from January 2007 to August 2008. Inclusions, but not the screening, were stopped when exactly fifteen patients were included in the respective groups. The antiplatelet drugs were withdrawn 5 days before surgery.

### Setting

The Pitié-Salpêtrière Vascular Surgical Register is a comprehensive prospective database containing the clinical and surgical characteristics of all patients undergoing vascular surgery at our institution since 1984. For this analysis the data coding was systematically verified. All patients who underwent infra-renal aortic reconstructive surgery (for infrarenal aneurysm or aortic stenosis) during a 18-month period were considered for this analysis. The protocol was approved by our institutional ethics committee (Comité de protection des personnes “Ile-de-France VI”) and the patients gave their written informed consent for participation and genotype analysis.

The inclusion criteria were as follows: continuous single-agent antiplatelet therapy for more than 28 days, scheduled major aortic surgery (for aortic stenosis or aneurysm), and an anaesthetic consultation at least 6 days before surgery in order to organize a 5-day interruption of antiplatelet therapy. The exclusion criteria were endoprosthetic procedures, congestive heart failure (NYHA 3–4), no antiplatelet treatment interruption, renal failure (creatinine clearance <30 mL/min) and dual-agent antiplatelet therapy.

### Perioperative Management

The patients were screened as recommended by the American College of Cardiology/American Heart Association Task Force [12]. Patients with poor or non-evaluable functional status, unstable coronary heart disease, or positive noninvasive myocardial stress testing underwent coronary angiography. Surgery was performed under general anesthesia with intravenous propofol, sufentanil and atracurium, as previously described [13], [14], [15]. A single preoperative dose of cefazolin (1.5 g) was administered one hour before the beginning of surgery in all patients. Perioperative management of chronic cardiovascular treatments (except antiplatelet agents) was conducted as previously described [15], [16], [17].

### Endpoints

We considered an effective antiplatelet treatment for patients taking aspirin based on arachidonic acid-induced platelet aggregation of less than 20% [18]. A previous study of healthy volunteers showed mean platelet aggregation of 70.8% (±18.5%) in response to arachidonic acid [19].

Clopidogrel efficacy was routinely measured in terms of ADP-induced platelet aggregation. An elevated risk of ischemic events has been reported in clopidogrel-treated patients who have residual aggregation values above 50–60% in response to 20 µM ADP in platelet-rich plasma [20]. Likewise, in diabetic patients, residual aggregation above 62% in response to 20 µM ADP was associated with the highest incidence of major cardiac events during long-term follow-up [21]. We therefore considered that residual aggregation below 50% in response to 20 µM ADP was associated with a good pharmacodynamic response to clopidogrel. Another way of assessing clopidogrel effectiveness is flow cytometry quantification of vasodilator-stimulated phosphoprotein (VASP) phosphorylation, which explores the P2Y12 receptor signaling pathway and is therefore theoretically more specific of clopidogrel effect than platelet aggregation [22].

The chosen platelet reactivity index (PRI) cut-off was 50%, in agreement with published data [23], [24].

Global platelet function was evaluated by collagen-induced platelet aggregation and by platelet–leukocyte complexes (PLC). PLC were enumerated to reflect the level of circulating activated platelets, as they are known as a sensitive biomarker of platelet activation, and were analyzed as a continuous variable.

### Blood Collection and Measurements

Venous blood (13 mL) was collected in Vacutainer tubes containing 0.105 M citrate (1 vol/9 vol) (BD Vacutainer, Becton Dickinson, Le Pont de Claix, France) at four time points:

– *D-5:* Blood was collected during the preoperative examination, when the treated patients had been on uninterrupted antiplatelet therapy for more than 28 days. Blood was not collected from patients in the control group at this time.
– *Day of surgery (Ds):* Blood was collected in the operating room, via a venous line, from patients in all three groups, immediately prior to surgery.
– *Two days after surgery:* blood was collected on the hospitalization ward the day of aspirin or clopidogrel resumption.
– *Seven days after surgery (D7):* blood was collected on the hospitalization ward five days after aspirin or clopidogrel resumption.

### Functional Platelet Assessment

Aggregation studies were performed within 3 hours after blood collection, on a TA-8V optical platelet aggregometer (Soderel Medical, Heillecourt, France). Platelet-rich plasma (PRP) was obtained by centrifugation at 200 *g* for 10 min, and the platelet count was adjusted to 250×10^9^/L. Aggregation tests were performed as described elsewhere [19], using the following agonists: arachidonic acid 1 and 2 mM (Helena biosciences Europe, Saint-Leu La Forêt, France), Horm collagen 1 µg/ml (Nycomed Pharma, Paris, France) and ADP 10 and 20 µM (Biopool, Umea, Sweden). Platelet aggregation was recorded for 8 minutes and expressed as maximal aggregation (%).

VASP (vasodilator-stimulated phosphoprotein) was measured in whole blood with a commercial kit (Platelet VASP; Diagnostica Stago, Biocytex, Asnières, France) on a FACScan flow cytometer (Becton Dickinson). The results were expressed as the platelet reactivity index (PRI, %), calculated as recommended by the manufacturer.

### Platelet-leukocyte Complexes

Whole-blood platelet-leukocyte complexes (PLC) were counted by flow cytometry. An aliquot of fresh whole citrated blood (500 µL) was fixed for 10 min at room temperature with 0.5% paraformaldehyde (PFA), then 20 µl of fixed blood diluted with 20 µL of Isoton diluent (Beckman Coulter, Roissy-Charles de Gaulle, France) was incubated with 4 µL of a phycoerythrin (PE)-conjugated anti-human-CD41 mAb (antiplatelet GPIIb receptor, Caltag, Burlingame, USA) and 4 µL of a FITC-conjugated anti-human-CD45 mAb (Beckman Coulter) to label the whole leukocyte population, for 15 min at room temperature in the dark. Then 500 µL of Isoton diluent containing 0.5% PFA was added and flow cytometry was performed within 4 hours. Nonimmune mAbs of the same isotype as the immune mAbs and provided by the same manufacturer were used as controls to determine the negative cut-off for each antibody.

The samples were analyzed with a FACScalibur device (Becton Dickinson). A threshold was set on CD45-positive events and 5000 leukocytes were acquired. PLC were identified as the cell population stained with both anti-CD41-PE and anti-CD45-FITC and were expressed as a percentage of total leukocytes.

### Genotyping

Genomic DNA was extracted from blood leukocytes, using standard techniques. CYP2C19*2 (681G>A; rs4244285) was genotyped using TaqMan Validated SNP assays (C_25986767_70) and a 7900HT sequence detection system (Applied Biosystems, Courtaboeuf, France).

### Statistical Analysis

Data are expressed as means ± SD and medians [95% confidence interval] for non-normally distributed variables (normality was assessed with the Shapiro-Wilk W test), or numbers (percentages). Mean values were compared using Student’s *t* test, with paired comparisons when appropriate. When more than two means were compared, analysis of variance was used, with repeated measures when appropriate. Because the wide dispersion of the values could be an issue in some cases, we used two additional indicators of distribution, namely the range and the coefficient of variability (100×standard deviation/mean, expressed as a percentage).

All P values are two-tailed, and P values of less than 0.05 were considered to denote significant differences. Statistical analysis was performed with SPSS software version 17 (SPSS Inc., Chicago, IL).

## Results

### Subjects

**Figure 1:**
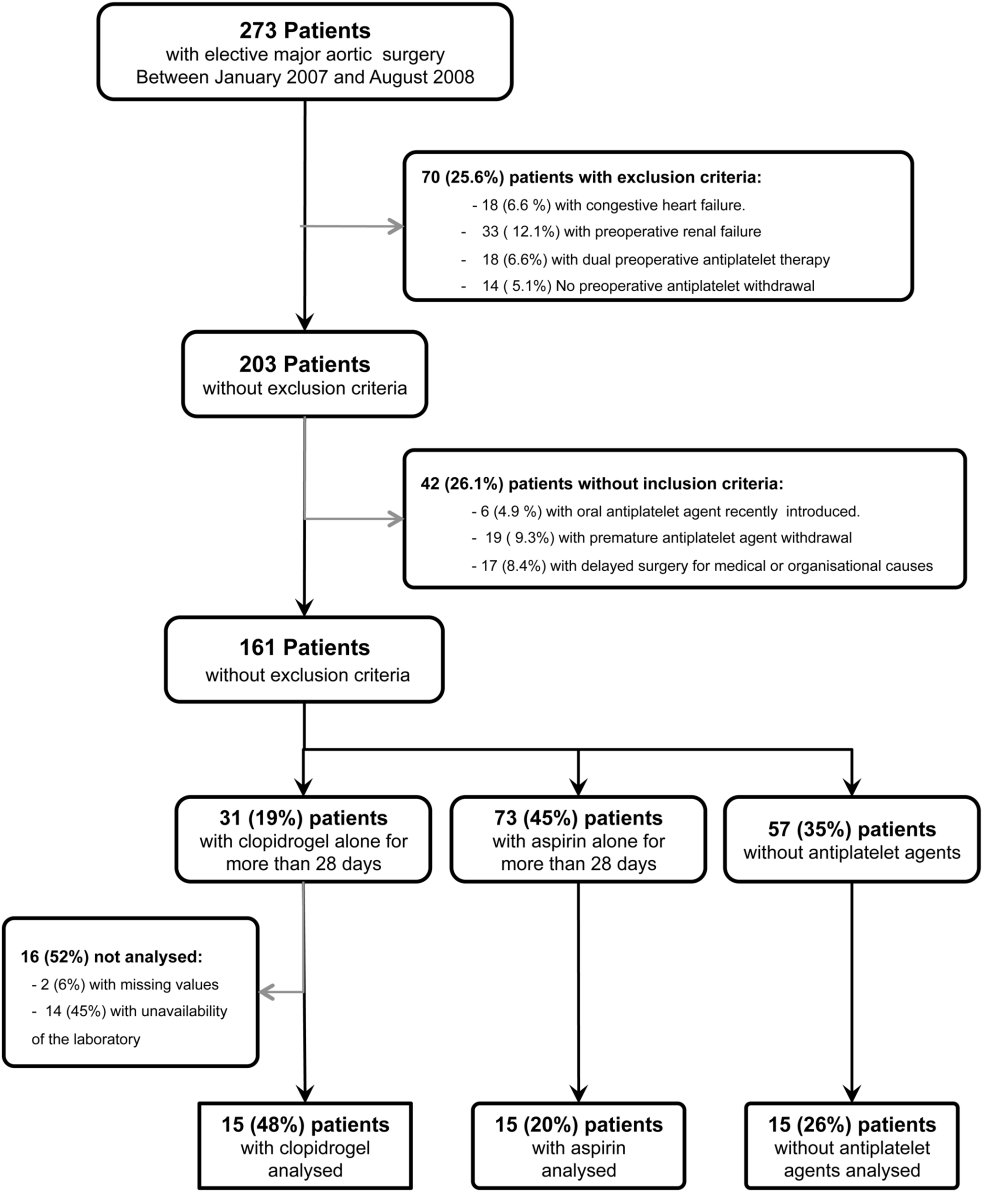
Flowchart of the study.

Consecutive patients fulfilling inclusion criteria were enrolled during a 18-month period among 273 patients scheduled for abdominal aortic reconstruction. As summarized in Figure 1, 45 patients (16.5% of those initially screened) were enrolled. As expected, coronary artery disease was more frequent in the patients taking aspirin or clopidogrel than in the controls (Table 1, p = 0.01). However, the predicted risk of postoperative cardiac complications (RCRI stratification) did not differ across the three groups (p = 0.15).

**Table 1:** Patients’ characteristics.

Variables	All Patients(n = 45)	AspirinGroup(n = 15)	ControlGroup(n = 15)	Clopidogrel Group(N = 15)		p Value
**Demographic characteristics**						
Age (yrs)	67±11	65±9	65±11	67±10		0.59
Male	36 (77)	16 (87)	9 (60)	14 (83)		0.18
**Medical history**						
Prior myocardial infarction	9 (19)	5 (33)	0 (0)	4 (24)	0.06	
Coronary revascularisation	7 (15)	4 (27)	0 (0)	3 (18)	0.11	
Coronary Artery Disease	11 (23)	7 (47)	0 (0)	4 (24)	0.01	
Heart Failure	2 (4)	2 (13)	0 (0)	0 (0)	0.11	
Hypertension	26 (55)	10 (67)	5 (33)	11 (65)	0.11	
Chronic obstructive pulmonary disease	18 (38)	4 (27)	7 (47)	7 (38))	0.51	
Pulmonary Failure	2 (4)	1 (7)	1 (7)	0 (0)	0.55	
Renal Failure	3 (6)	1 (7)	1 (7)	1 (6)	0.99	
Diabetes	7 (15)	3 (20)	2 (13)	2 (12)	0.79	
**Surgical characteristics**						
Aneurysm	21 (45)	8 (53)	8 (53)	5 (29)		0.28
Combined Surgery	7 (15)	1 (7)	2 (13))	4 (24)		0.40
**RCRI stratification**						
1	29 (62)	6 (40)	12 (80)	11 (65)		
2	14 (30)	6 (40)	3 (20)	5 (30)		0.15
≥3	4 (8)	3 (20)	0 (0)	1 (6)		
**Preoperative medications**						
ACE inhibitors	21 (44)	8 (53)	5 (33)	8 (47)		0.53
Beta-blockers	15 (32)	9 (60)	1 (7)	5 (29)		0.01
Nitrates	3 (6)	3 (20)	0 (0)	0 (0)		0.03
Calcium Blockers	11 (23)	6 (40)	0 (0)	5 (29)		0.03
Diuretic	10 (21)	2 (13)	4 (27)	4 (23)		0.64
Anti-Arrhythmic	1 (2)	0 (0)	0 (0))	1 (6)		0.41

Data are presented as the mean value ± SD or number (%) of subjects. RCRI: Revised Cardiac Risk Index; ACE: angiotensin converting enzyme. CI = confidence interval.

### Impact of Antiplatelet Treatment Interruption on Platelet Functions

#### Control Group

On the day of surgery, controls showed mean aggregations of 62%±22% and 66%±13% in response to 2 mM arachidonic acid and 20 µM ADP, respectively. These values were of 62%±21% and 74%±15% at D7.

Platelet aggregation induced by a low collagen concentration (1 µg/mL) was 45%±31% On the day of surgery (Ds, representing the baseline for the control group) and 67%±20% on D7 (p = 0.03), and consistent with the previous reports about the platelet hyperaggregability observed after major vascular surgery.

#### Aspirin Group

All the patients treated with aspirin received a 75 mg/d to 81 mg/d maintenance dose.

**Figure 2:**
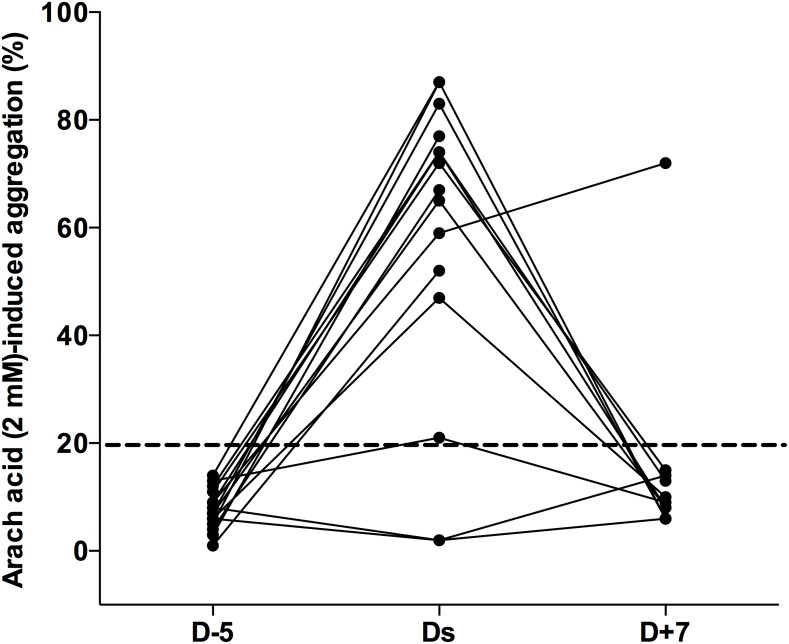
Impact of aspirin withdrawal and reintroduction on arachidonic acid-induced platelet aggregation. D-5: baseline value determined during preoperative examination. Ds: day of surgery. D+7: seven days after surgery, five days after aspirin resumption Arachidonic acid was used at 2 mM. Platelet aggregation is expressed as maximal aggregation (%).

At baseline (D-5), the platelet response to arachidonic acid was below 20%, with little inter-individual variability (mean 7.5%±2.8%, CV 38%, range 9%). On Ds, 5 days after aspirin interruption, the response to arachidonic acid exceeded 20% in all but two of the patients (Figure 2). The mean absolute increase in the response was 45%±29% (p<0.0001 compared to 0%). Two days after surgery, results did not differ (p>0.1, data not shown) but the two patients with an aggregation level below 20% on the day of surgery had recovered normal values. On D7, response to arachidonic acid again showed little inter-individual variability (mean 15%±17%, CV 108%; range 61%). Values did not differ significantly between D-5 and D7 (mean difference: 7.8%±16.3%; p = 0.13 compared to 0%). However, large differences between D-5, Ds and D7 were noted in three patients (Figure 2). Post-study investigations showed that aspirin interruption was doubtful in two cases and that aspirin had not been correctly reintroduced postoperatively in one case.

Collagen-induced platelet aggregation was 28%±11% immediately prior to aspirin withdrawal (D-5), a value significantly lower than the control baseline value (p<0.0001), in line with the impact of the thomboxane pathway inhibition on the collagen-induced aggregation. Platelet aggregation was normalized on Ds (49%±28%, p = 0.77 versus the control baseline value) and on D+2 (67±11%, p NS versus the control baseline value). No difference was observed on D7 compared to D-5 (1%±25%; CV 2500, range 97%; p = 0.86), whereas value was significantly lower than in the control group on D7 (29%±20% versus 67%±20%, p<0.001).

#### Clopidogrel Group

All the patients treated with clopidogrel received a 75 mg/d maintenance dose.

**Figure 3:**
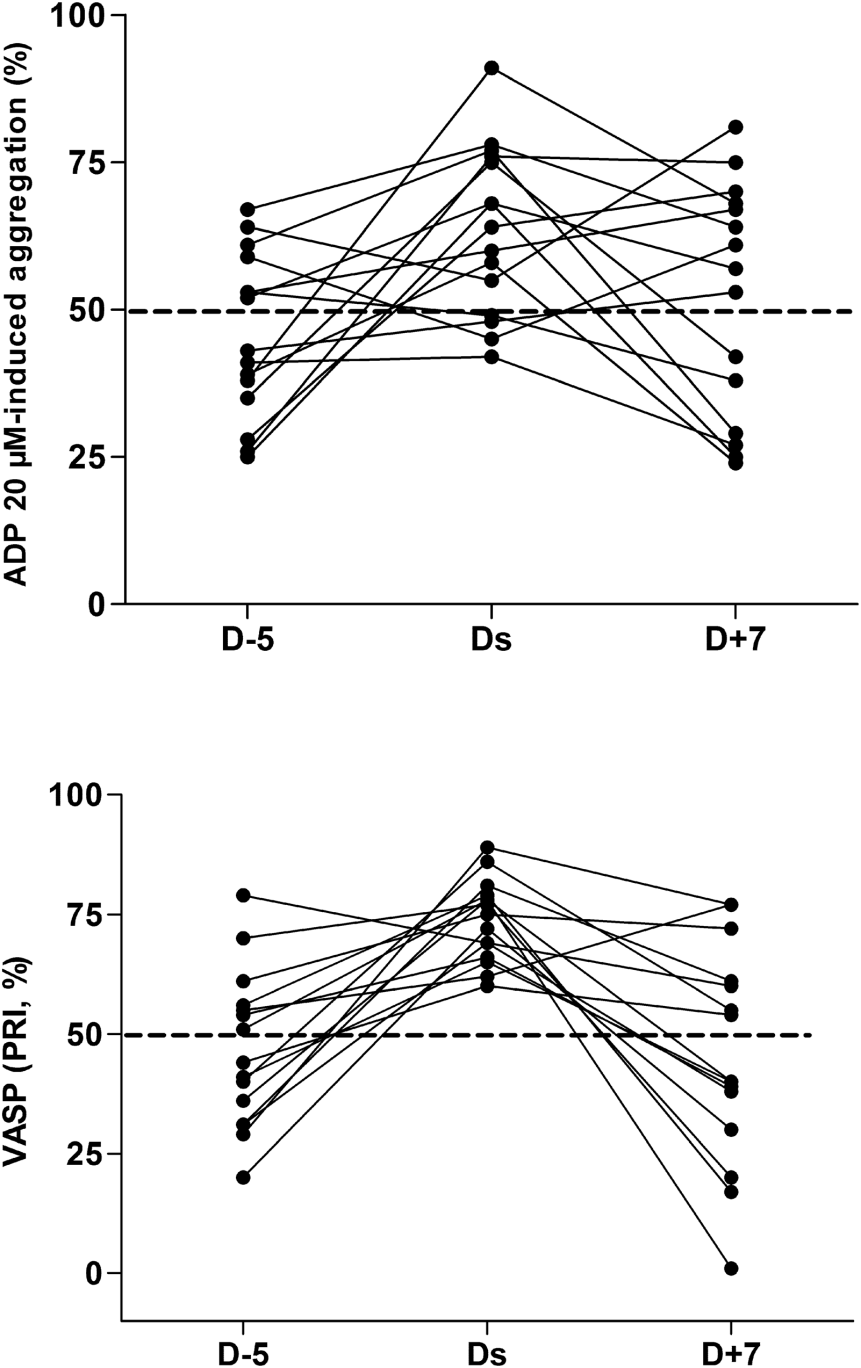
Impact of clopidogrel withdrawal and reintroduction on platelet functions. A: Platelet aggregation response to 20 µM ADP, expressed as maximal aggregation (%). B: VASP phosphorylation level, expressed as the Platelet Reactivity Index (%). D-5: baseline value; Ds: day of surgery; D+7: seven days after surgery, five days after clopidogrel 75 mg resumption.

On D-5, the platelet response to ADP 20 µM showed, as expected, large inter-individual variability (mean 46%±13%, CV 28%; range 32%). Five days after clopidogrel interruption, only 11 patients had recovered an ADP platelet response of more than 50% (Figure 3A). The mean absolute increase was 17%±20% (p = 0.004 compared to 0%). Two days after surgery, results did not differ (p>0.1, data not shown). On D7 the ADP response also showed large inter-individual variability (mean 52%±20, CV 40%; range 57%). Values differed significantly between D-5 and D7 (mean difference 12%±22%; p = 0.04 compared to 0%), suggesting that clopidogrel reintroduction for five days was insufficient to attain optimal platelet function inhibition in these patients. Similar results were obtained with 10 µM ADP (data not shown). Poor clopidogrel responders at baseline were tested for the CYP2C19*2 variant, and all were negative [25].

Using VASP assay, 9 clopidogrel-treated patients (60%) were good clopidogrel-responders with VASP levels below 50% on D-5 (Figure 3B). On Ds, 5 days after clopidogrel withdrawal, all 15 patients in this group had values above 50%. The mean absolute increase in VASP was 27%±20% (CV 75%; range 71%; p<0.0001 compared to 0%). On D7, seven patients (33%) had VASP values above 50%, of whom five also had ADP-induced aggregation above 50%. No significant difference in VASP values between D-5 and D7 was noted (1.1%±29%; P = 0.89 compared to 0%), probably because of the marked inter-individual variability (CV 2640%; range 99%).

Collagen-induced aggregation was 65%±10% in the clopidogrel-treated patients at baseline (p = 0.57 compared to the control baseline), and did not vary on Ds or D7 (respectively 66%±16% and 58%±19%, p = 0.45 versus D-5). No significant difference was observed relative to the control group on D7 (58%±19% versus 67%±20%, p = 0.20).

### Impact of Antiplatelet Treatment Interruption on Platelet-leukocyte Complexes

**Figure 4:**
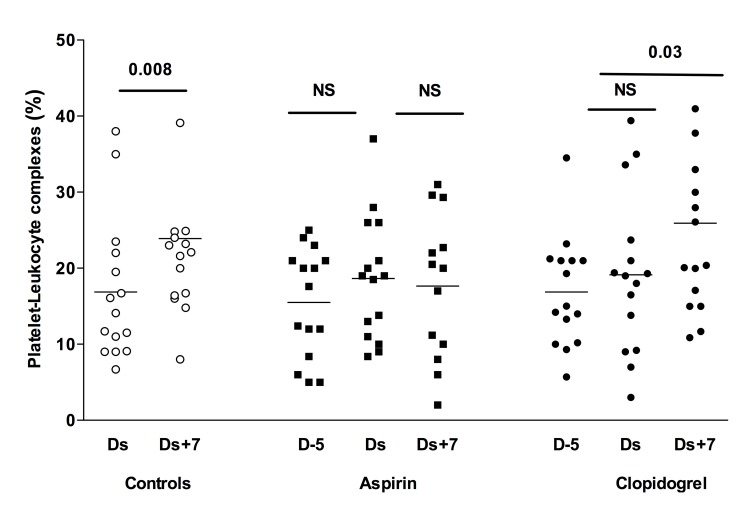
Platelet-Leukocyte Complex (PLC) levels in the three patient groups. PLC are expressed as a percentage of total leukocytes. D-5: baseline value for aspirin and clopidogrel groups. Ds: day of surgery, baseline value for the control group. D+7: seven days after surgery, five days after aspirin or clopidogrel 75 mg resumption.

Platelet-leukocyte complexes (PLC) as measured by flow cytometry reflect the number of activated platelets that express P-selectin, thereby binding to the leukocyte PSGL receptor. At baseline, PLC numbers were similar in the three groups (control 17%±9%; aspirin 14%±7%; clopidogrel: 16%±7%; p = 0.57) (Figure 4). On the day of surgery, PLC numbers were not significantly higher than on D-5 in the aspirin or clopidogrel group (respectively +6±10%, p = 0.08; and +3±9%, p = 0.23). A significant increase in PLC numbers was observed in the control group and in the clopidogrel group on D7 compared to baseline (respectively +7%±11, p = 0.03; and +10%±15, p = 0.02), whereas the increase in the aspirin group was not significant (+4%±10, p = 0.16).

## Discussion

First finding in this study is that most aspirin-treated patients had recovered normal platelet aggregation on the day of surgery, 5 days after aspirin withdrawal, and that inter-individual variability was low at baseline, after aspirin withdrawal, and after aspirin reintroduction. No significant increase in platelet-leukocyte complex levels occurred after aspirin reintroduction. Such complexes have been described as a sensitive marker of platelet activation in acute coronary syndromes and after cardiac surgery [26], and correlate strongly with the risk of clinical thrombosis [27], [28], [29].

A second finding is the considerable inter-individual variability of the clopidogrel response, at baseline, 5 days after withdrawal, and after reintroduction. It should be noted that none of the patients had the loss-of-function CYP2C19*2 polymorphism, which affects clopidogrel biotransformation into its active metabolite [25]. Importantly, PLC levels were elevated 5 days after clopidogrel reintroduction, in line with an ineffective platelet inhibition.

On the day of surgery, platelet aggregation was near-normal in the aspirin group, suggesting that drug withdrawal five days prior to surgery is sufficient to normalize the platelet functions. This is in line with an another study performed in healthy volunteers and in patients [30]. Moreover, PLC levels were similar to those in the control patients, who were free of cardiovascular risk factors, suggesting the absence of abnormal platelet activation leading to a potential thrombotic risk or a rebound phenomenon. Further studies are required to determine whether aspirin can be safely withdrawn less than five days before surgery. The mean PLC level measured five days after aspirin reintroduction was lower than the contemporary control value, suggesting that this schedule of perioperative aspirin management is not associated with the postoperative hyperaggregability described in similar patients [31], and observed here in the control group. These results support early aspirin reintroduction after major vascular surgery, but this must be confirmed in studies with clinical endpoints (late postoperative bleeding or thrombotic complications). In other situations, such as elective non-cardiac surgery, whether aspirin should be maintained or not during the perioperative period remains an unsolved issue. Indeed, the STRATAGEM study did not find any difference in terms of occurrence of major thrombotic or bleeding events between preoperative maintenance or interruption of aspirin in 291 patients treated with aspirin for secondary prevention [32]. Recently published POISE2 results confirmed these observations in 10010 patients [9].

The results in the clopidogrel group raise several issues. The main concern is the well-known high degree of inter-individual variability at baseline such as observed in various clinical situations [33], end even higher five days after drug withdrawal, as previously observed after a 600-mg loading dose [34]. While clopidogrel withdrawal five days before surgery appeared adequate in most cases, some patients had incomplete platelet recovery in terms of platelet aggregation. These results are in line with those obtained by our group in healthy volunteers [25]. Furthermore, and conversely to what observed with aspirin, the PLC remained significantly increased 5 days after clopidogrel reintroduction at the 75 mg daily maintenance dose on day 2 after surgery, suggesting that the timing was suboptimal. Further studies are required to determine whether clopidogrel reintroduction should start with a bolus, or whether earlier reintroduction provides a better balance between the risks of bleeding and thrombosis. Finally, in view of the strong variability of the clopidogrel responses in this population, it might be better to use a more predictable antiplatelet drug in the perioperative period, such as ticagrelor. Alternatively, aspirin might be added to clopidogrel in the early post-operative period.

This study has several limitations. First, the treatment allocation was not randomized, raising the possibility that the effects observed here might be influenced by baseline differences across the three arms. However, no design included randomization was possible to test the predefined hypotheses, and the study population was too small for multivariate analysis. Among the potential confounders, proton pump inhibitors and statins may have affected the response to antiplatelet drugs and the platelet reactivity. Also, the analyses were conducted on an intent-to-treat basis, and might have been affected by poor adherence to the protocol. Indeed, two patients in the aspirin group did not stop taking aspirin as planned, while aspirin was not reintroduced postoperatively in one case. However, exclusion of these three patients would have enhanced the observed differences across the groups or the time points. Lastly, the use of surrogate markers to quantify platelet inhibition and potential clinical risks is questionable, but these biomarkers are considered robust in various clinical situations. In particular, patients with poor platelet responses to clopidogrel are at an increased risk of cardiovascular events [35]. In addition, treatments with better clinical antithrombotic efficacy, such as prasugrel, have a stronger inhibitory effect on platelet functions (aggregation and VASP) but also increase the hemorrhagic risk [36]. No clear correlation has been established between the bleeding risk and platelet function. A working group of the European Society of Cardiology [37] has concluded that further studies are required before defining platelet reactivity cut-offs associated with better or worse clinical outcome. We used arbitrary cut-offs but also report the results as continuous variables.

### Conclusion

Aspirin withdrawal 5 days before high-bleeding-risk procedures appears to be adequate to restore platelet function. In contrast, clopidogrel withdrawal at the same time point is associated with highly variable platelet recovery. Likewise, aspirin reintroduction on day 2 yielded an expected effect on day 7, but this was not the case for clopidogrel. Further studies are required to refine clopidogrel management before and after hemorrhagic surgery.
